# Molecular Genetics and Pathogenesis of the Floating Harbor Syndrome: Case Report of Long-Term Growth Hormone Treatment and a Literature Review

**DOI:** 10.3389/fgene.2022.846101

**Published:** 2022-05-18

**Authors:** Mariia E. Turkunova, Yury A. Barbitoff, Elena A. Serebryakova, Dmitrii E. Polev, Olga S. Berseneva, Elena B. Bashnina, Vladislav S. Baranov, Oleg S. Glotov, Andrey S. Glotov

**Affiliations:** ^1^ Federal State Budget Institution of Higher Education “North-Western State Medical University Named After I.I Mechnikov” Under the Ministry of Public Health of the Russian Federation, Saint-Petersburg, Russia; ^2^ Department of Genomic Medicine, D.O.Ott Research Institute of Obstetrics, Gynaecology and Reproductology, St. Petersburg, Russia; ^3^ Bioinformatics Institute, St. Petersburg, Russia; ^4^ Department of Genetics and Biotechnology, St. Petersburg State University, St. Petersburg, Russia; ^5^ City Center for Medical Genetics, St. Petersburg, Russia; ^6^ Children’s Scientific and Clinical Center for Infectious Diseases of the Federal Medical and Biological Agency, St. Petersburg, Russia; ^7^ Laboratory of Biobanking and Genomic Medicine of Institute of Translation Biomedicine, St. Petersburg State University, Saint-Petersburg, Russia

**Keywords:** Floating Harbor syndrome, SRCAP, growth hormone therapy, short stature, whole-exome sequencing, facial dysmorphisms

## Abstract

**Introduction:** Floating Harbor syndrome (FHS) is an extremely rare disorder, with slightly more than a hundred cases reported worldwide. FHS is caused by heterozygous mutations in the *SRCAP* gene; however, little is known about the pathogenesis of FHS or the effectiveness of its treatment.

**Methods:** Whole-exome sequencing (WES) was performed for the definitive molecular diagnosis of the disease. Identified variants were validated using Sanger sequencing. In addition, systematic literature and public data on genetic variation in *SRCAP* and the effects of growth hormone (GH) treatment was conducted.

**Results:** We herein report the first case of FHS in the Russian Federation. The male proband presented with most of the typical phenotypic features of FHS, including short stature, skeletal and facial features, delayed growth and bone age, high pitched voice, and intellectual impairment. The proband also had partial growth hormone deficiency. We report the history of treatment of the proband with GH, which resulted in modest improvement in growth prior to puberty. WES revealed a pathogenic c.7466C>G (p.Ser2489*) mutation in the last exon of the FHS-linked *SRCAP* gene. A systematic literature review and analysis of available genetic variation datasets highlighted an unusual distribution of pathogenic variants in *SRCAP* and confirmed the lack of pathogenicity for variants outside of exons 33 and 34. Finally, we suggested a new model of FHS pathogenesis which provides possible basis for the dominant negative nature of FHS-causing mutations and explains limited effects of GH treatment in FHS.

**Conclusion:** Our findings expand the number of reported FHS cases and provide new insights into disease genetics and the efficiency of GH therapy for FHS patients.

## 1 Introduction

Floating Harbor syndrome (FHS, OMIM 136140) is an extremely rare autosomal dominant disease. The name of the disease originates from the names of the two hospitals (Boston Floating Hospital in Massachusetts and at Harbor General Hospital in California) in which the first two cases of FHS were reported in the 1970s (Pelletier and Feingold (1973); Leisti et al. (1975)). FHS is characterized with typical facial dysmorphologies, anomalies of bone development, speech, intellectual disability, and abnormal growth features (Pelletier and Feingold (1973); Leisti et al. (1975); Patton et al. (1991); Lacombe et al. (1995)). Several dozen FHS patients have so far been reported worldwide, with 24 different causal genetic variants discovered (Son et al. (2020)).

The genetic cause of FHS are heterozygous mutations in the *SRCAP* gene. A peculiar property of all reported pathogenic variants in this gene is their unique clustering in the exons 33 and 34 of the gene, which we will discuss in greater detail later (Seifert et al. (2014)). The *SRCAP* gene encodes the main catalytic subunit of the multi-protein SNF2-Related CBP Activator Protein (*SRCAP*) chromatin remodeling complex (Hood et al. (2012)). In the vast majority of cases, *de novo* mutations in *SRCAP* are identified in FHS patients. Autosomal dominant inheritance of the disease was described in two families with mother-to-daughter transmission (White et al. (2010); Arpin et al. (2012)).

One of the main phenotypic manifestations of FHS is the short stature and the delay in bone age, though only a few patients with efficient growth hormone treatment have been reported (Son et al. (2020); Seifert et al. (2014); Galli-Tsinopoulou et al. (2011); García et al. (2012); Homma et al. (2019); Stagi et al. (2007)). FHS patients frequently display low levels of stimulated somatotropin (STH), likely influencing the development of a short stature (Galli-Tsinopoulou et al. (2011); Cannavò et al. (2002)). In several research efforts, low efficiency of GH treatment has been reported, and the selection of efficient therapy for FHS patients remains complicated and requires further investigation (Nagasaki et al. (2014)). In this work, we describe the clinical history and treatment of the first FHS patient in Russian Federation during the course of 15 years. We also review recent literature on FHS genetics and provide additional guidelines for sequencing data interpretation in FHS-like patients.

## 2 Case Description

### 2.1 Ethical Statement

The study was conducted in accordance with the Ethics Committee of the Federal State Budget Institution of Higher Education “North-Western State Medical University named after I.I Mechnikov”, extract from minutes No. Four dated 04/04/2018. The patient gave written informed consent to participate in the study. The study was performed in accordance with the Declaration of Helsinki.

### 2.2 Early Disease Manifestation and Clinical History of the Proband

The proband is the second child in a family. The proband was born by a full-term normal spontaneous delivery with the birth weight of 3,250 g, the birth length of 50 cm. The proband has normal 46 XY karyotype.

At the age of 9 months the proband was first consulted by an endocrinologist because of growth delay. At this age, thyroid status of the proband was investigated, with no thyroid pathology found. Since the earliest age the patient was carefully observed by specialists in the fields of neurology and ophthalmology, with the following additional symptoms being recorded: organic brain lesions, delayed speech development, multiple stigmas of dysembryogenesis and strabismus.

At the age of 3 the proband was exposed to a complex endocrinological assessment. At this age, the proband was characterized by a significant growth delay (height of the proband - 80.5 cm, standard deviation score (SDS) = -3.83), height velocity 4 cm/year (SDS = -2.93). Upon comprehensive assessment no dysfunction of either thyroid or adrenal gland was found. Bone age of the proband was estimated as 8–9 months. Growth hormone stimulation test with clonidine showed the maximum STH release level of 7.2 ng/ml. No pathological changes in hypophyseal-hypothalamic brain regions were discovered using MRI. Considering) these results, the proband was diagnosed with partial somatotropin deficiency and growth hormone treatment was recommended. Since the age of 4 years and 6 months, the patient received somatotropin therapy. No adverse effects of the treatment have been reported, except for high IGF-1 levels.

**FIGURE 1:**
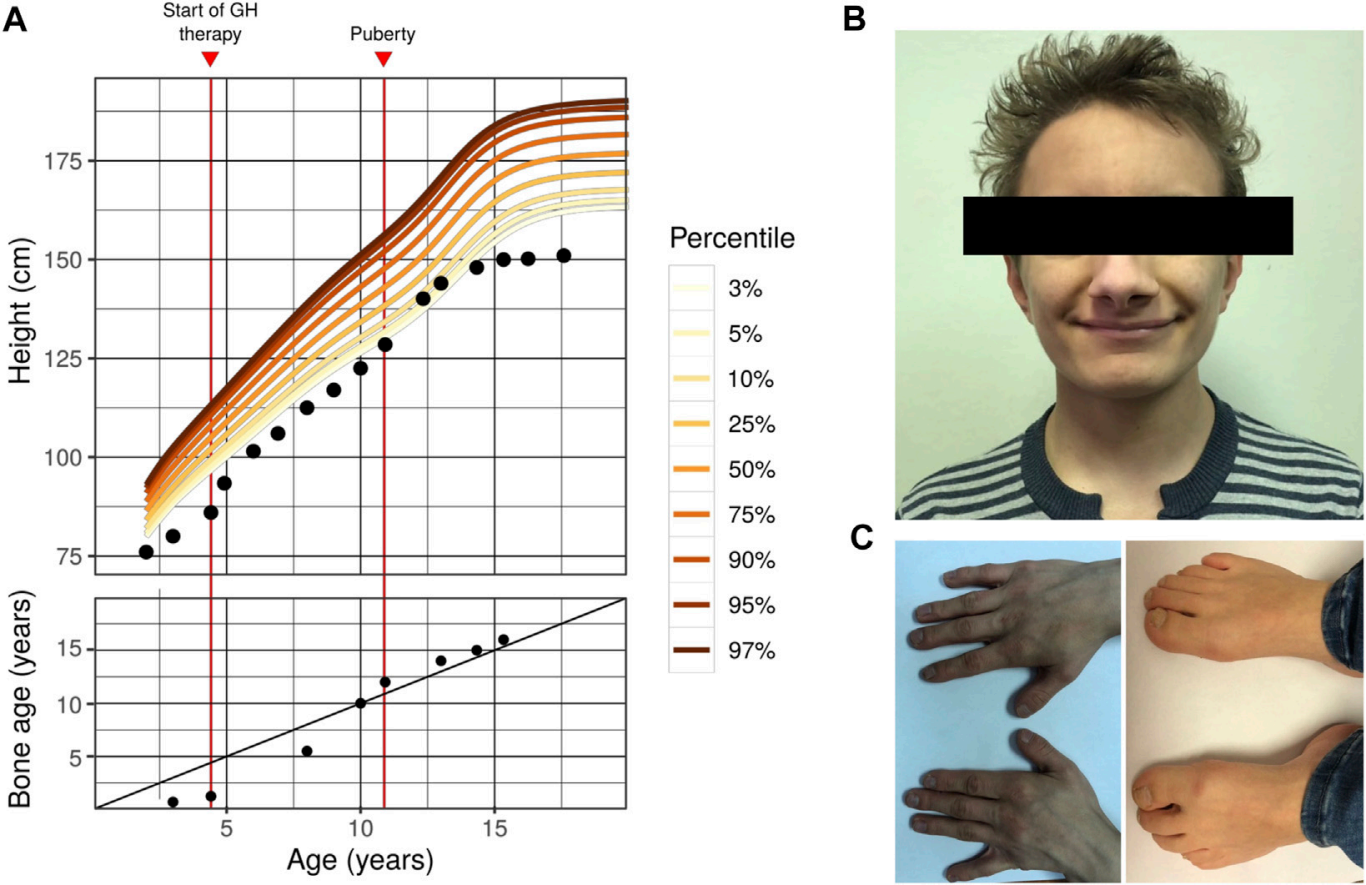
Phenotype of the first FHS patient in Russian Federation **(A)** Growth curve (top) and bone age dynamics (bottom) of the proband with Floating-Harbor syndrome. Colored lines represent the corresponding percentile of the reference male growth curve (data collected from https://www.cdc.gov/growthcharts/percentile_data_files.htm). On the bottom plot, the diagonal line represents 1:1 correspondence between actual age and bone age **(B)** A photo showing facial dysmorphisms of the proband **(C)** Photos showing the proband’s hands (left) and feet (right). Typical skeletal features of FHS might be seen (bilateral brachyphalangy of the thumb; shortening of the fourth and fifth toes). Other typical features of our patient include: high-pitched voice; delayed speech development (his speech is composed of a limited set of simple words); intellectual disability; strabismus; growth and bone age delays).

All the growth dynamics data is represented in Table 1. A substantial growth delay can be seen (notised), which is concordant with a typical case of FHS (comparison of the normal growth curve and the growth of the proband is presented in Figure 1A). Height of the parents is normal: 163 cm (mother), 183 cm (father), the target proband height (mother’s height + father’s height±13 cm/2) = 173 ± 6.5 cm. Patients with Floating-Harbor syndrome typically display a wide range of phenotypic features, including facial and skeletal abnormalities, speech and language delay, growth and bone age delays. At the age of 17, the patient was characterized with the majority of aforementioned phenotypic features. These phenotypic abnormalities are described below.

**TABLE 1:** Anthropometrics, endocrinological and radiological parameters recorded for proband at different ages.

Age (years, months)	Height (cm)	Height SDS	Weight (kg)	BMI SDS	IGF-1 (mg/ml)	Bone age
At birth	50		3.2			
1 year	71	-1.97				
2 years	76	-3.3				
3 years	80	-3.7	9.5	-0.91		8–9 months
4 years 5 months (beginning of GH treatment)	86	-4.11	11	-0.62		1 year 3 months
4 years 11 months	93.4	-3.05	12.7			
6 years	101.5	-2.55				
6 years 11 months	106	-2.58	16.4	-0.73		
8 years	112.5	-2.39	18.3	-0.93		5 years 6 months
9 years	117	-2.44	21	-0.42		
10 years	122.5	-2.29	23.1	-0.61		10 years
10 years 11 months (puberty)	128.5	-1.95	28.1	0.11	134.7 (-1 SDS)	12 years
12 years 4 months	140.1	-1.24	33.5			
13 years	144	-1.2	36.8	-0.12		14 years
14 years 4 months	148	-1.79	40	-0.25	577 (+1 SDS)	15 years
15 years	149.7	-2.2	41.4	-0.37		
15 years 4 months (switch to the metabolic GH dose)	150	-2.49	41.9	-0.40	652 (+2 SDS)	16 years
16 years 3 months (end of treatment)	150.2	-3.28	41.2			
17 years 7 months	151	-3.53	43.8		540 (+1 SDS)	

### 2.3 Facial Abnormalities

Facial abnormalities are one of the key phenotypic features of FHS (Robinson et al. (1988)). These dysmorphisms include triangular face, narrow bridge of the nose with broad nose tip and big nostrils, low *columella* position, horizontal and broad mouth fold, thin vermilion, phialine lower lip, deep-set eyes with antimongoloid slant, long eyelashes, big low set ears. Nearly all of the aforementioned features are present in the phenotype of the proband (Figure 1B), with the exception of big ears and long eyelashes. In addition to typical features, strabismus was observed in our proband.

### 2.4 Skeletal Abnormalities

FHS is characterized by a number of differences in the skeletal structure (especially, hands and legs), possibly due to abnormal differentiation and proliferation of chondrocytes (Nagasaki et al. (2014)). Hand and arm abnormalities include clinodactyly or brachydactyly of the thumb. These clinical features are similar to those seen in the Rubinstein-Taybi syndrome (RTS), the similarity apparently stems from the similarity in the molecular genetics pathology between FHS and RTS (the genetic cause of RTS is mutations in the *CREBBP* gene that is functionally linked to *SRCAP*). FHS patients might have the following leg abnormalities: absence of nails in both first toes, broad first toes and brachydactyly, clubbing, prominent joints, and big toes. Short neck and additional pair of ribs have been described in FHS. In our proband the typical FHS features of arms and legs such as bilateral brachyphalangy of the thumb, shortening of the fourth and fifth toes are noticed (Figure 1C), though no short neck or additional rib pair are found.

### 2.5 Height and Bone Age

FHS patients typically display progressive short stature. The delay in prenatal development is frequently observed (Son et al. (2020); García et al. (2012); Homma et al. (2019)). As detailed previously, height and weight of our proband at birth were normal; and pronounced growth delay and partial growth hormone deficiency were detected in childhood. Bone age of our proband had long been significantly below the actual age (e.g., at age of eight the proband’s bone age was 5 years and 6 months), with quick progression seen after puberty. Such changes are also typical for FHS (Nikkel et al. (2013)). In some of the earlier cases, gonadotropin-releasing hormone (GnRH) agonists were used to slow down puberty (e.g., in Galli-Tsinopoulou et al. (2011)); however, such treatment was not used in our patient due to a lack of molecular diagnosis until the patient turned 16. The height SDS before the GH treatment was -4.11. After completing the therapy the height SDS was -3.53.

### 2.6 Speech and Other Features

Floating-Harbor syndrome is commonly characterized by specific features of speech, such as high-pitched voice and, in some patients, nasal voice. One of the main phenotypic features of FHS is a significant speech development delay with pronounced imperfect articulation of speech primarily due to verbal dyspraxia (White et al. (2010)). Structural abnormalities in the neck have been reported in FHS, surgical correction of such abnormalities has shown to improve speech development in FHS patients (White et al. (2010); Homma et al. (2019)). The degree of speech disorder may vary from patient to patient, with severe cases leading to inability of verbal communication. Our proband has a high-pitched voice; his speech is composed of a limited set of simple words. Apart from typical speech features, FHS patients generally display intellectual disability. Similar intellectual issues were observed in our proband; the proband was educated at a special school.

Several additional phenotypic features were reported for FHS, including nystagmus, strabismus, hearing problems and deafness, choanal atresia, multiple dental caries, malocclusion, heart and kidney development abnormalities, malabsorption syndrome, cryptorchism, hypothyreosis, epilepsy etc. (Nikkel et al. (2013); Milani et al. (2018)). No such traits were seen in our proband.

## 3 Materials and Methods

### 3.1 Medical Procedures

The patient has been monitored by a specialized hospital for children with short stature of various etiology since the age of 3 years. Standard growth measurement techniques were used. Short stature was defined as height SDS < -2. Growth SDS calculations were performed using the Auxology software. Expected height of the patient was calculated from parents’ height using a standard procedure. Blood hormone levels (TTH, free thyroxine (T4), cortisol, prolactin, and IGF-1) were measured. To diagnose growth hormone deficiency, standard stimulation test was performed as follows: the proband was perorally administered with clonidine (0.15 *μ*g/kg), blood STH levels was assessed 15 min before clonidine administration, as well as at 0, 15, 30, 60, 90, and 120 min after. Growth hormone deficiency was diagnosed if peak STH levels were below 7 ng/ml; peak STH levels between 7 and 10 ng/ml was considered as evidence of partial growth hormone deficiency. Bone age was estimated based on the results of radiological analysis of the proband’s hands. Additionally, the patient was subjected to the ultrasound scan of the thyroid gland, abdominal organs, and kidneys; brain MRI with a focus on hypophysis was performed.

Growth hormone treatment was conducted by administering 0.030–0.035 mg/kg per day by subcutaneous injections in the evening of each day during the course of the therapy.

### 3.2 Whole-Exome Sequencing

For whole-exome sequencing, peripheral venous blood samples of the proband were collected in EDTA, and DNA was extracted with a QIAsymphony automated station for the isolation of nucleic acids and proteins. 1 *μ*g of purified DNA in 1x Low TE buffer (pH = 8.0) was used as a starting material and sheared on Diagenode BioRuptor UCD-200 DNA Fragmentation System to the average DNA fragment size of 170–180 bp. The shearing conditions were as follows: L-mode, 50 min of sonication cycles consisting of 30 s sonication and 30 s pause. Library preparation and exome capture were performed using SeqCap EZ MedExome Kit (Roche, USA) following the SeqCap EZ Library SR User’s Guide, v5.1 without modification. DNA libraries were amplified using seven PCR cycles, and 14 PCR cycles were performed for amplification of enriched libraries. Library quality was evaluated using QIAxcel DNA High Resolution Kit on QIAxcel Advanced System. Libraries were sequenced using 2x151 bp paired-end reads using Illumina HiSeq 4,000 sequencer.

### 3.3 Bioinformatic Data Analysis

Sequencing reads were aligned onto the b37 human reference genome assembly using bwa mem v. 0.7.15-r1140 (Li and Durbin (2009)). Alignment files were pre-processed, and variants were called using Genome Analysis ToolKit (GATK) v. 3.5.0 (Van der Auwera et al. (2013)). Variant calling and genotyping was performed in cohort (-ERC GVCF) mode. Variants were filtered using Variant Quality Score Recalibration (VQSR) with truth sensitivity thresholds of 99.9 (for SNPs) and 99.0 (for indels). Filtered variants were annotated using SnpEff/SnpSift (Cingolani et al. (2012)) with the following reference databases: 1,000 Genomes phase 3 (Auton et al. (2015)); Exome Aggregation Consortium (Lek et al. (2016)); in-house Russian exome allele frequencies (Barbitoff et al. (2019); Shikov et al. (2020)), as well as NCBI ClinVar and dbNSFP v 2.9 (Li and Durbin (2009)). Custom software was used for enhanced variant interpretation.

### 3.4 Sanger Sequencing

To validate the identified candidate variants, Sanger sequencing of the proband’s DNA, as well as his parents and sister (having no FHS symptoms) was performed. To this end, we designed a custom pair of primers (TTC​CTG​CCC​TTG​TTC​CTG​TC (forward) and CCA​CAG​CAA​CTG​GCA​ACA​GAT (reverse)) to amplify the corresponding DNA fragment that was then subjected to sequencing using the ABI 3500X platform. Presence of the mutation was confirmed by visual inspection of the sequencing chromatograms.

## 4 Results

### 4.1 Whole-Exome Sequencing Identifies a Pathogenic c.7466C>G Variant in *SRCAP*

As detailed in the previous sections, our proband had all the major phenotypic features characteristic of FHS. However, to obtain the definitive molecular diagnosis of the disease we performed whole-exome sequencing of the proband at the age of 16 years (see Methods for details). A total of 37,985 short variants (SNPs and indels) inside targeted exome regions were identified. Of these, high-impact (nonsense-, splice site, and frameshift) variants were selected, totaling 481 variants. Additional filtering was applied based on the allele frequency in the general population (using reference datasets such as 1,000 Genome (Auton et al. (2015)), ExAC (Lek et al. (2016)), and in-house Russian exome database (Barbitoff et al. (2019); Shikov et al. (2020))). Out of the remaining variants, the NM_006,662.2 c.7466C>G (p.Ser2489*) variant in the 34th exon of the *SRCAP* gene was identified in the heterozygous state. Sanger sequencing of the corresponding region in proband, his parents and sibling showed that the variant is a *de novo* mutation that is not present in mother, father, or sister (Supplementary Figure S1). The identified variant has previously been reported as likely pathogenic in a study by Zhang et al. (2019). According to the new evidence, we can now classify the variant as pathogenic according to the American College of Medical Genetics and Genomics (ACMG) guidelines for variant interpretation (Richards et al. (2015)). The variant in our case matches the following ACMG criteria: PVS1 (null variant in a causal gene), PS2 (*de novo* nature confirmed), PS4 (variant previously reported in unrelated cases with similar phenotype (Zhang et al. (2019))), PM1 (location in a known mutational hot spot), PM2 (absent from control populations), PP5 (reputable source recently reports variant as pathogenic, but the evidence is not available to the laboratory to perform an independent evaluation (SCV001433564)). As thus, we can conclude that our proband can be diagnosed with the Floating-Harbor syndrome, increasing the number of reported cases of this extremely rare genetic disorder.

### 4.2 Analysis of *SRCAP* Variation Identifies Numerous Likely Benign pLoF Variants

**FIGURE 2:**
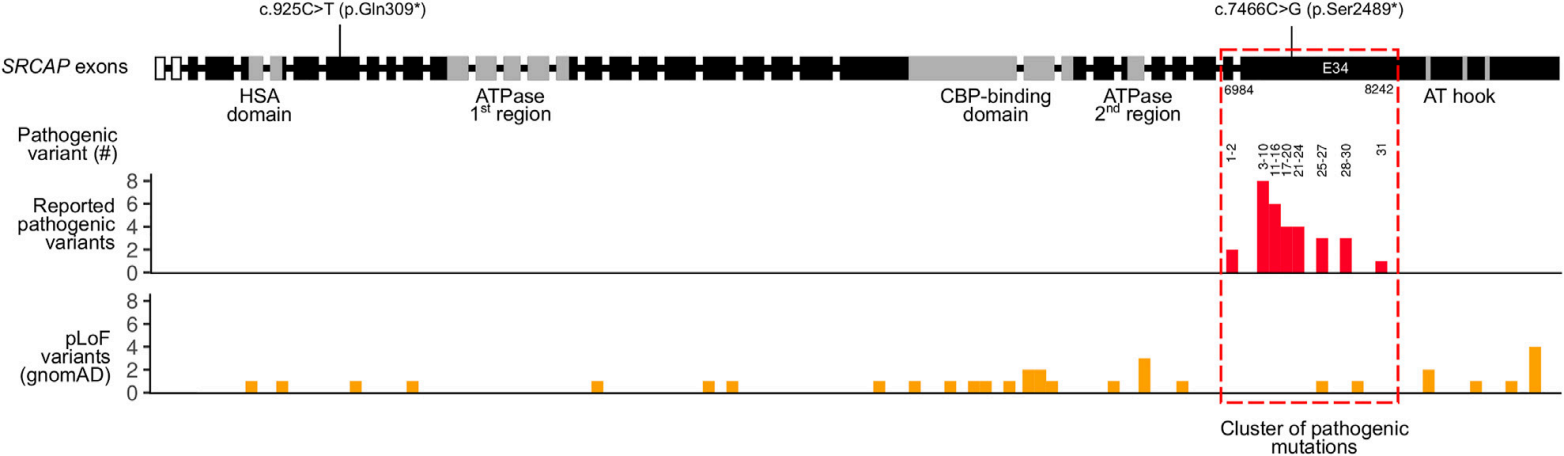
Summary of pathogenic and putative loss-of-function (pLoF) variants in the *SRCAP* gene. Intron-exon structure of the *SRCAP* gene is shown on top according to the NM_006,662.2 RefSeq transcript (intron sizes are not preserved). Boundaries of *SRCAP* protein domains are drawn according to UniProt and Hood et al. (2012). Locations of a likely pathogenic c.7466C>G (p.Ser2489*) variant found in an FHS individual in this case, as well as a benign c.925C>T (p.Gln309*) variants found in healthy Russian controls are shown. Reported pathogenic variants’ coordinates from Table 2 are represented in a histogram below. Numbers on top of the histogram correspond to the number of a pathogenic variant in Table 2. For gnomAD variants, both gnomAD v. 2.1.1 and gnomAD v. 3.1 data were used.

As mentioned previously, known pathogenic variants in *SRCAP* are clustered in the exons 33 and 34 of the gene (Table 2). The same localization is observed for our pathogenic c.7466C>G (p.Ser2489*) variant identified in the proband. Given such an unusual clustering of pathogenic mutations in the last exons of the *SRCAP* gene in FHS patients, we next questioned if variants located upstream of this exon have any effect on the phenotype and FHS-like manifestation. To answer this question, we leveraged publicly available genome variation datasets such as the Genome Aggregation Database (gnomAD) (Karczewski et al. (2020)). We selected putative loss-of-function (pLoF) variants in *SRCAP* (nonsense mutations, splice region, and frameshift variants without any additional flags) from gnomAD v.2.1.1 and v. 3.1 datasets. Such selection resulted in a set of 33 variants with allele frequencies ranging from 4 × 10^−6^ to 8 × 10^−5^ (Figure 2). Almost all of the variants present in gnomAD control individuals are located upstream and downstream of the main hotspot of reported pathogenic variants in *SRCAP* (Figure 2; Table 2). This finding further corroborates the hypothesis about the benign nature of pLoF variants in *SRCAP* located outside the start of the exon 34. Additionally, we used our in-house database of Russian exome allele frequencies (Barbitoff et al. (2019); Shikov et al. (2020)) to search for novel pLoF variants located in *SRCAP* gene in non-FHS patients. Indeed, we observed one variant, c.925C>T (p.Gln309*), which occured two times in our dataset of 1,292 non-FHS samples, amounting to a lower boundary of minor allele frequency = 4 × 10^−4^. As this novel variant was observed solely in non-FHS individuals, we can conclude that this variant, as well as other pLoF *SRCAP* variants present in gnomAD, are likely benign and have no relationship to FHS.

**TABLE 2:** FHS-causing mutations in *SRCAP* described in literature.

No	Mutation position	Protein consequence	References
1	c.6985C>T	p.Arg2329*	Seifert et al. (2014)
2	c.7000C>T	p.Gln2334*	Kehrer et al. (2014)
3	c.7165G>T	p.Glu2389*	Arpin et al. (2012); Nikkel et al. (2013)
4	c.7218dupT	p.Gln2407Serfs*36	Seifert et al. (2014)
5	c.7218_7219delTC	p.Gln2407fs*35	Hood et al. (2012); Nikkel et al. (2013)
6	c.7219C>T	p.Gln2407*	Nikkel et al. (2013)
7	c. 7227dupA	p.Ala2409fs	Homma et al. (2019)
8	c.7229dupA	p.Asn2410Lysfs*33	Le Goff et al. (2013)
9	c.7230insA	p.Asn2410fs*32	Nikkel et al. (2013)
10	c.7245_7246delAT	p.Ser2416ArgfsTer26	Zhang et al. (2019)
11	c.7262dupG	p.Arg2421fs	Homma et al. (2019)
12	c.7274insC	p.Thr2425fs*17	Nikkel et al. (2013)
13	c.7275_7276delAC	p.Pro2426Thrfs*16	Nikkel et al. (2013)
14	c.7303C>T	p.Arg2435*	Son et al. (2020)
15	c.7316dupC	p.Ala2440fs*3	Son et al. (2020)
16	c.7330C>T	p.Arg2444*	Son et al. (2020)
17	c.7374dupT	p.Pro2459fs*125	Nikkel et al. (2013)
18	c.7376delC	p.Pro2459fs*16	Nikkel et al. (2013)
19	c.7394delC	p.Pro2465Glnfs*10	Milani et al. (2018)
20	c.7395delA	p.Val2466Tyrfs*9	Seifert et al. (2014)
21	c.7466C>G	p.Ser2489Ter	Zhang et al. (2019), this work
22	c.7533_7534insAA	p.Thr2512fs*5	Nikkel et al. (2013)
23	c.7534_7535insAA	p.Thr2512Lysfs*11	Nikkel et al. (2013)
24	c.7549delC	p.Gln2517fs*5	Son et al. (2020)
25	c.7684G>T	p.Glu2562*	Homma et al. (2019)
26	c.7732dupT	p.Ser2578Phefs*6	Choi et al. (2018)
27	c.7736_7737delTT	p.Leu2579Argfs*4	Le Goff et al. (2013)
28	c.7851dupC	p.Asn2618Glnfs*12	Nikkel et al. (2013)
29	c.7852insC	p.Asn2618fs*11	Nikkel et al. (2013)
30	c.7863dupG	p.Gln2622fs	Le Goff et al. (2013)
31	c.8242C>T	p.Arg2748*	Nikkel et al. (2013)

## 5 Discussion

To date, pathogenesis of short stature and delayed bone maturation is not completely understood. Some of the patients with these conditions have decreased plasma levels of IGF-1 with normal levels of STH release in stimulation tests. These data suggest that some neuroendocrine defects likely cause decreased spontaneous growth hormone secretion and, as a result, lead to low levels of circulating GH in patients (Stagi et al. (2007); Aimaretti et al. (2000)). Some patients with short stature have been diagnosed with somatotropin deficiency in stimulation tests (García et al. (2012); Homma et al. (2019); Nikkel et al. (2013)); at the same time, effects of GH treatment in such children are variable; moreover, GH therapy frequently resulted in increased levels of IGF-1, suggesting additional deficiency in IGF-1 signaling (García et al. (2012); Homma et al. (2019)).

Floating-Harbor syndrome is one of the genetic conditions leading to short stature combined with a variety of phenotypic abnormalities (see above). The genetic cause of FHS are mutations in the *SRCAP* gene that encodes the main catalytic subunit of an SNF2-Related CBP Activator Protein (SRCAP) chromatin remodeling complex. This complex plays a crucial role in the incorporation of an H2A.Z histone variant in nucleosomes and serves as a co-activator of CREB-binding protein, thus regulating activity of a multitude of genes (Johnston et al. (1999); Luk et al. (2010)). Despite a clear involvement of *SRCAP* in the growth processes, it is yet unclear, however, which part of the GH-IGF-1 signaling axis is perturbed in FHS. It has been hypothesized that FHS phenotype is caused by defects in signaling downstream of IGF-1 and not by a decrease in either GH or IGF-1 secretion and/or activity itself. This hypothesis is supported by previous studies that showed limited GH efficacy in FHS (Son et al. (2020)).

According to the results published in 2019, height SDS in FHS patients after GH treatment increased to -2.26 ± 0.8 compared to -4.1 ± 1.2 in patients that did not receive GH therapy (Homma et al. (2019)). In our study, we describe a patient who received GH therapy in growth doses (0.030–0.035 mg/kg) for over 11 years. During the course of the therapy, elevation of the patient’s IGF-1 levels have been observed multiple times, not allowing to increase the dose of GH. The final recorded height of the patient was 151 cm (SDS = -3.53), and no adverse effects of GH therapy have been reported. In other studies, the outcomes of GH therapy (where reported) varied. In two FHS patients, final height SDS ranged from -2.5 to -1.2 who also received treatment for preliminary puberty (Homma et al. (2019)). In a German study, the final height SDS of FHS patient receiving GH therapy was -1.8; at the same time, patients who did not receive GH therapy had an average height SDS of -3.7 with closed growth plates (Seifert et al. (2014)). Japanese researchers reported a case of an incomplete GH therapy, in which GH administration was terminated due to poor response after 2 years of the treatment. Height SDS of this patient at the age of 14 was -3.6 (Nagasaki et al. (2014)). In Italian study, a patient receiving GH therapy combined with gonadoliberin agonists showed a final height SDS of -1.2 (Stagi et al. (2007)). We summarized the data on the final (adult) height of patients with genetically confirmed FHS on and without GH therapy given in Table 3. Out of five patients who received the GH treatment for whom the adult height has been reported, only two displayed final adult height SDS <3. Taken together, these results do not allow to unequivocally draw a conclusion regarding the extent to which GH therapy alleviates FHS effects on height. However, it seems that the effects of GH treatment on FHS are at best modest, indicating that the main molecular pathology in FHS is not caused by decreased GH secretion or activity.

**TABLE 3:** Summarized data on the final adult growth and treatment of GH in patients with geneticists confirmed FHS. ^†^ - patient treated with a GnRH agonist.

No	*SRCAP* mutation	Gender	Age of GH treatment start	Duration of GH treatment (dose mg/kg/day)	Height, cm	SDS height at start	Adult height, cm	SDS adult height	References
1	c.7303C>T (p.Arg2444*)	M	10 years	2 years (0.033)	99.6	-4.9	137	-3.6	Nagasaki et al. (2014)
2	c.7218dupT (p.Gln2407Serfs*36)	F	9 years	5 years	NA	NA	154	-1.8	Seifert et al. (2014)
3	c.7303C>T p.(Arg2435*)	M	10 years	2 years	NA	NA	154.5	-3.3	Menzies et al. (2020)
4^†^	c.7330C>T (p.Arg2444*)	F	10 years	4 years 1 month (0.05)	NA	NA	147	-2.5	Homma et al. (2019)
5	c.7466C>G (p.Ser2489*)	M	4 years 6 months	11 years (0.033)	86	-4.11	151	-3.5	Present study
6	c.7330C>T (p.Arg2444*)	F	Not treated	-	NA	NA	140	-3.7	Seifert et al. (2014)
7	c.7275_7276delAC p.(Pro2426Thrfs*16)	F	Not treated	-	NA	NA	139.8	-3.6	Menzies et al. (2020)
8	c.7227dupA (p.Ala2409fs)	M	Not treated	-	NA		141	-3.5	Homma et al. (2019)
9	c.7684G>T (p.Glu2562*)	M	Not treated	-	NA	NA	150	-3	Homma et al. (2019)
10	c.7303C>T (p.Arg2435*)	M	NA	NA	NA	NA	145.5	-4.1	Hood et al. (2012)
11	c.7303C>T (p.Arg2435*)	M	NA	NA	NA	NA	148	-3.8	Hood et al. (2012)

In addition to short stature, FHS patients frequently display delayed bone age; however, the delay disappears near the age of puberty (around 10 years) (Nikkel et al. (2013)). In our case, puberty occurred at the age of 10 years and 11 months and progressed rapidly, as did the process of bone maturation. Several cases with precocious puberty in FHS have been reported; some of the patients received treatment with gonadotropin-releasing hormone agonists (Galli-Tsinopoulou et al. (2011); Homma et al. (2019); Stagi et al. (2007)). We believe that the short stature of FHS patients may be (at least part) linked to the rapid progression of puberty and bone maturation.

We have systematically reviewed all published studies describing the spectrum of identified mutations in FHS patients. These studies include patients from various countries, including the United States (Homma et al. (2019)), China (Zhang et al. (2019)), South Korea (Son et al. (2020)) Japan (Nagasaki et al. (2014)), and others. The results of this analysis are presented in Table 2. Notably, the vast majority of reported pathogenic and likely pathogenic variants fall inside the exon 34 of the *SRCAP* gene that corresponds to the fragment of the protein directly preceding the AT-hook. Such a clustering of pathogenic variants has been discussed previously (Hood et al. (2012); Zhang et al. (2019)), though several patients with mutations outside of exon 34 (Kehrer et al. (2014)) or with no mutations in the coding part of *SRCAP* (Le Goff et al. (2013)) have been reported. It has been suggested that the FHS-causing mutations have a dominant negative effect, with the corresponding truncated protein variants lacking an important regulatory region in the C-terminus. It has also been noted that the complete loss of the *SRCAP* protein function has no pathogenic effect as deletion of the entire *SRCAP* gene has been reported in a HapMap individual with no reported FHS-like phenotype.

In concordance with these data, a substantial number of putative loss-of-function (pLoF) variants have been reported in healthy individuals from gnomAD in nearly all parts of the *SRCAP* gene except for the region that has the highest enrichment of pathogenic variants (Figure 2). Most of the gnomAD pLoF variants were observed once across gnomAD and the more common variants (for example, c.1135–2A > G splice acceptor variant occuring 17 times across gnomAD) still had a minor allele frequency (MAF) of <1 × 10^−4^. While the very presence of such variants in gnomAD individuals suggests their likely benign nature, their possible role in disease can not be confidently ruled out. However, in addition to the gnomAD pLoF variants in *SRCAP*, we also discovered a common pLoF allele that was specific to the Russian Exome dataset (Barbitoff et al. (2019); Shikov et al. (2020)). The c.925C>T (p.Gln309*) variant was observed 2 times in a sample of several hundred non-FHS individuals and can be classified as a benign variant according to the ACMG Standards and Guidelines for variant interpretation (Richards et al. (2015)). Altogether, the presence of a high-frequency Russian-specific pLoF variant in the eighth exon of the gene, as well as the high total frequency of gnomAD pLoF variants in *SRCAP*, suggests that, indeed, truncating variants that are located before exons 33 and 34 are not pathogenic and should not be interpreted as having a pathogenic effects in FHS individuals in spite of the current guidelines. It is also important to note that several variants at the very end of exon 34 are also present in gnomAD (Figure 2), suggesting that even variants that fall inside exon 34 but outside the pathogenic mutation hotspot, do not cause FHS. At the same time, a recent report by Rots et al. (Rots et al. (2021)) suggested that proximal *SRCAP* variant might cause another neurodevelopmental disorder, characterized by an altered DNA methylation pattern. However, high frequency of proximal pLoF variants in *SRCAP* suggests that such a phenotype should be much more common than FHS and/or have incomplete penetrance.

**FIGURE 3:**
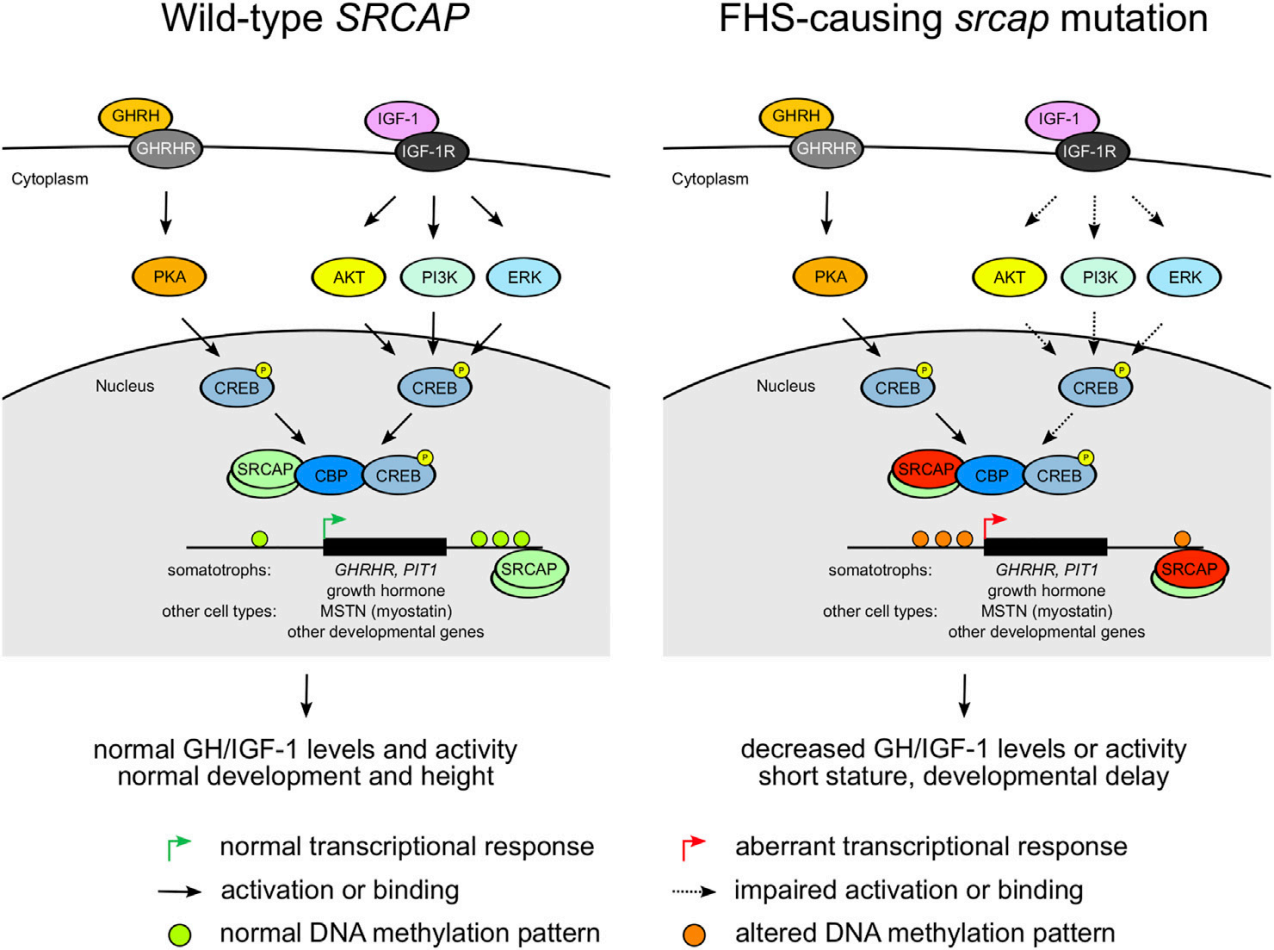
A diagram showing potential pathogenetic mechanisms underlying the Floating Harbor syndrome. Dashed arrows indicate impaired activation or binding.

All the studies of FHS genetics emphasize the importance of variants in exons 33 and 34 for FHS. It has been suggested that the truncated SRCAP protein variants act in a dominant negative manner (Messina et al. (2016)) and escape nonsense-mediated decay (Rots et al. (2021)). However, it remains unclear how the genetic changes observed in FHS patients are connected to the phenotype. Such understanding may enhance our ability to find efficient therapeutics for this disease. Given the aforementioned phenotypic similarity between RTS (caused by *CREBBP* mutations) and FHS, we might hypothesize that the ability of the SRCAP protein to interact with CBP (and thus regulate CREB activity) plays a central role in the pathology of both FHS and RTS. Given the localization of FHS-causing mutations, it is likely that mutant SRCAP is capable of binding to CBP and CREB. However, the regulation of the SRCAP-CBP interaction is likely to be altered, which may result in the aberrant transcription of CREB-CBP targets. Importantly, cAMP signaling and CREB play a role in both GH production (Bertherat (1997)) and cellular response to IGF-1 in various cell types (Zheng and Quirion (2006); Zuloaga et al. (2013)). Hence, expression of multiple CREB target genes related to growth and development (e.g., PIT-1 transcriptional regulator or myostatin,, a key regulator of muscle growth (Zuloaga et al. (2013))) is likely to be altered in FHS. These effects are complemented by a general effect of FHS-causing mutations on DNA methylation pattern (Rots et al. (2021)). Taken together, all these factors may drive the phenotypic manifestation of FHS (Figure 3). However, the exact molecular mechanism behind FHS requires further experimental investigation.

The *SRCAP* gene provides an excellent example of the challenges for automated variant prioritization. In many prioritization strategies, pLoF variants in disease genes are generally considered as having high impact on the phenotype, with an exception for pLoF sites in the last exon. In *SRCAP*, however, variants in the last exons seem to be almost exclusively related to FHS phenotype, while truncating variants in other parts of the gene (and even at the very end of exon 34) are not pathogenic in the context of FHS. Hence, new sophisticated computational approaches are needed for accurate prediction of pLoF variants’ pathogenicity in genes where loss-of-function is not a major disease mechanism at play.

## Data Availability

The datasets for this article are not publicly available due to concerns regarding participant/patient anonymity. Requests to access the datasets should be directed to the corresponding author.
