# Supplementary material for: Molecular Genetics and Pathogenesis of the Floating Harbor Syndrome: Case Report of Long-Term Growth Hormone Treatment and a Literature Review
Source: Front Genet. 2022 May 18;13:846101. doi: 10.3389/fgene.2022.846101 (PMC9157637; doi:10.3389/fgene.2022.846101)
Supplement: Supplementary file 1 [file DataSheet1.pdf]

Supplementary Material

1 SUPPLEMENTARY FIGURES

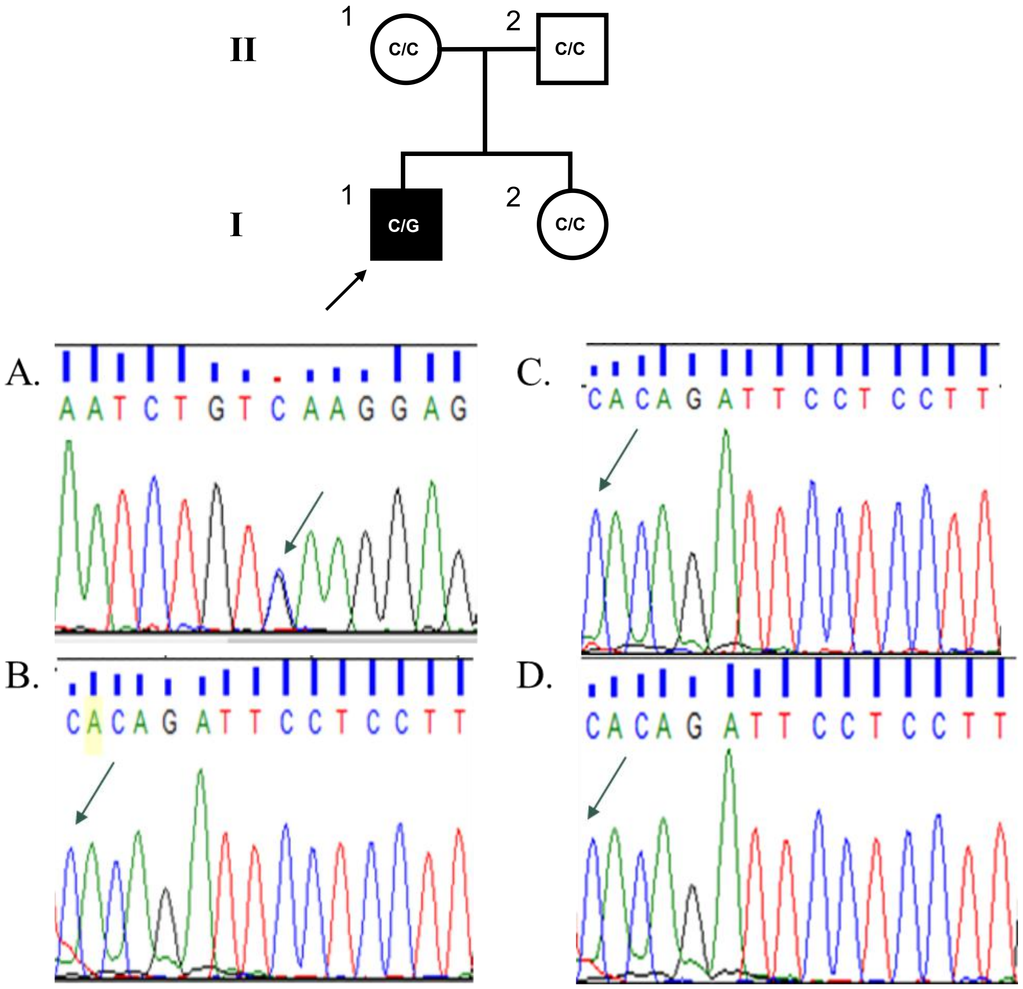

Figure S1. Validation of the identified pathogenic c.7466C¿G (p.Ser2489*) variant. Top, pedigree of the family. Arrow indicates the proband. Roman numbers correspond to generations. Bottom, Sanger squencing results for the proban (A) and the unaffected family members: father (B), mother (C), and sibling (D). Arrows indicate variant position. Please note that chromatogram on (A) was obtained using the forward primer; chromatograms on (B-D) were obtained using the reverse primer. Matching chromatogram for the proband is not shown due to low sequencing quality.
